# Investigation of early antibiotic use in pediatric patients with acute respiratory infections by high‐performance liquid chromatography

**DOI:** 10.1002/bmc.4699

**Published:** 2019-11-12

**Authors:** Pham Van Toi, Khanh V. Doan, Ngo Ngọc Quang Minh, Pham Nguyen Phuong, Menno D. de Jong, H. Rogier van Doorn, Thomas Pouplin

**Affiliations:** ^1^ Oxford University Clinical Research Unit, Wellcome Trust Major Oversea Programme Ho Chi Minh City—in Partnership with Hospital for Tropical Diseases Ho Chi Minh City Vietnam; ^2^ Department of Pharmacology, School of Medicine Tan Tao University Long An Vietnam; ^3^ Children's Hospital 1 Ho Chi Minh City Viet Nam; ^4^ Department of Medical Microbiology Academic Medical Centre Amsterdam The Netherlands; ^5^ Centre for Tropical Medicine and Global Health, Nuffield Department of Clinical Medicine University of Oxford Oxford UK; ^6^ Mahidol‐Oxford Tropical Medicine Research Unit, Faculty of Tropical Medicine Mahidol University Bangkok Thailand

**Keywords:** high‐performance liquid chromatography, *β*‐Lactams, antibiotic misuse, pediatric patients, urine

## Abstract

In this study, we developed and validated two reliable high‐performance liquid chromatography (HPLC) methods for the qualitative detection of six oral *β*‐lactams, which are commonly used in pediatric patients with acute respiratory infections (ARIs). Two distinct reverse‐phase chromatographic separations of six *β*‐lactams were obtained. Four *β*‐lactams (cefadroxil, cephalexin, cefaclor and cefixime) in urine were separated using a gradient program with a mobile phase consisting of K_2_HPO_4_ buffer (20 mm, pH 2.8) and acetonitrile on a LichroCART 250 × 4.6 mm, Purospher STAR C_18_ end‐capped (5 μm) column. Two remained *β*‐lactams (amoxicillin and cefuroxime) were analyzed using a gradient elution with the mobile phase containing K_2_HPO_4_ buffer (20 mm, pH 3.0) and acetonitrile on a LichroCart^®^ Purospher Star C_8_ end‐capped column (5 μm, 125 × 4.6 mm). Good linearity within the range of 0.3–30 μg/ml for cefadroxil, cephalexin, cefaclor and cefixime, and 0.2–20 μg/ml for amoxicillin and cefuroxime, was attained. The precisions were <14%. The accuracies ranged from 85.87 to 102.8%. The two validated methods were then applied to determine these six antibiotics in 553 urine samples of pediatric patients with ARIs. As a result, 32.2% were positive with one or more of six tested *β*‐lactams. Cefixime was the most commonly detected agent, accounting for 9.8% of enrolled patients.

## 1 INTRODUCTION

Antimicrobial resistance is a global concern and a particular pressure in developing countries, and is primarily attributed to the inappropriate use of antibiotics (Costelloe, Metcalfe, Lovering, Mant, & Hay, 2010; Goossens, Ferech, vander stichele, & Elseviers, 2005; Karakonstantis & Kalemaki, 2019; VNMOH, 2003). Unfortunately, the situation of self‐medication habits and the vast availability of oral antibiotics in drugstores has led to the misuse of antibiotics by patients and healthcare providers in Vietnam (Larsson et al., 2000). Acute respiratory infections (ARIs) are common illness in tropical countries, particularly in the pediatric population. Even though most ARI cases in children are viral infections, oral antibiotics are frequently prescribed, especially when viral or bacterial etiologies are indistinguishable (Do et al., 2011; van Gageldonk‐Lafeber et al., 2005). Hence, the misuse of antibiotics is a common practice in the community setting of ARI treatment in Vietnam and other countries (Hoa et al., 2009; Landers, Ferng, Mcloughlin, Barrett, & Larson, 2010; Llor & Cots, 2009).

Irrational and inappropriate use of antibiotics can be recorded from questionnaire‐based surveillance of the patients performed by healthcare providers during physical examination at admission (GARP, 2010; Jansen et al., 2006; Otters, van Der Wouden, Schellevis, van Suijlekom‐Smit, & Koes, 2004). However, in Vietnam, for most cases of ARIs requiring hospitalization, the reliability and objectiveness of the information about previous exposure to specific antibiotics provided by patients at admission are questionable and unverified. Identification of specific antibacterial agents used prior to hospitalization in these cases using an analytical method could be helpful to verify such information and help clinicians to choose a rational antibiotic regimen.

Among all oral antibiotics, the six *β*‐lactams (cefadroxil, cephalexin, cefaclor, cefixime, amoxicillin and cefuroxime) are the most commonly used antibacterial agents for the treatment of mild to moderate respiratory bacterial infections in both hospital and community settings in Vietnam (GARP, 2010). All of six *β*‐lactams are excreted at a high rate in urine in unchanged forms (AHFS, 2009), making urine a valuable and effective biological matrix for the qualitative or quantitative determination of antibiotics in patients (El‐Gindy, El Walily, & Bedair, 2000; Samanidou, Hapeshi, & Papadoyannis, 2003; Samanidou, Ioannou, & Papadoyannis, 2004). Many studies have described high‐performance liquid chromatography (HPLC) methods for the determination of oral cephalosporins in various matrices such as plasma (Mcateer, Hiltke, Silber, & Faulkner, 1987; Samanidou et al., 2003; Verdier et al., 2011; Wolff et al., 2013) and urine (Samanidou et al., 2003; Samanidou et al., 2004). In addition, Cazorla Reyes et al. also developed a liquid chromatography tandem mass spectrometry method for the analysis of 21 antibiotics including cephalosporins and various other antibiotics in plasma, urine, cerebrospinal fluid and bronchial aspiration matrix (Cazorla‐Reyes, Romero‐González, Frenich, Rodríguez Maresca, & Martínez Vidal, 2014). Although several HPLC methods have been developed, some are limited to quantification of one or two drugs (Bhinge, Malipatil, & Sonawane, 2014; Danafar, 2015; El‐Gindy et al., 2000; Khan et al., 2011), while others have been developed for the detection of 5–12 cephalosporins (Denooz & Charlier, 2008; Legrand, Vodovar, Tournier, Khoudour, & Hulin, 2016; Verdier et al., 2011; Wolff et al., 2013). In addition, many methods using liquid chromatography coupled to tandem mass spectrometric detection (LC/MS‐MS) were set up to measure simultaneously from five to 11 cephalosporins (Carlier, Stove, De Waele, & Verstraete, 2015; Colin, DE Bock, T'jollyn, Boussery, & Van Bocxlaer, 2013). Of note, several sample preparation procedures were applied to treat samples before injection into the HPLC systems: protein precipitation (Carlier et al., 2015; Khan et al., 2011; Legrand et al., 2016; McAteer et al., 1987; Samanidou et al., 2003; Verdier et al., 2011; Wolff et al., 2013), liquid–liquid extraction (Bhinge et al., 2014) and solid‐phase extraction (SPE) (Colin et al., 2013; Nemutlu, Kir, Katlan, & Beksac, 2009). A pre‐dilution step is commonly applied to treat urine samples because the high ionic strength of this matrix interferes with the analyte detection methods (Eshra, Hassan, & El–Walily, 1993; Kovach, Lantz, & Brier, 1991; Najib, Suleiman, El‐Sayed, & Abdulhameed, 1987; Samanidou et al., 2003; Samanidou et al., 2004). Additionally, some new extraction procedures have recently been applied to treat complex matrices aiming at reducing solvent consumption and the use of toxic solvents, in accordance with the Green Analytical Chemistry concepts. These innovative procedures also allowed analytical performance to be improved using modest and routine instrument configurations such as UV–vis detectors, while avoiding the use of more complex and expensive systems such as tandem mass spectrometers (Kabir, Locatelli, & Ulusoy, 2017). In the present study, two reliable HPLC with diode array detection methods were developed and validated for the detection of the six antibiotics in urine samples. Subsequently, the two distinct validated methods were applied on the samples collected at hospital admission from pediatric patients with ARIs who attended to the outpatient clinic of Children's Hospital 1, Ho Chi Minh City (CH1) to investigate the early use of these antibiotics in the community setting.

## 2 EXPERIMENTAL

### 2.1 Reagents and solutions

All reagents and solvents used were of analytical grade. Potassium dihydrogen phosphate (KH_2_PO_4_), di‐potassium hydrogen phosphate (K_2_HPO_4_), phosphoric acid (H_3_PO_4_), formic acid (HCO_2_H), HPLC‐grade acetonitrile (ACN) and methanol (MeOH) were purchased from Merck (Darmstadt, Germany). Water was purified by a Purelab UHQ system (ELGA, Marlow, UK). The reference standards of cefadroxil (CFD), cephalexin (CPL), cefaclor (CFO), cefixime (CFI), amoxicillin (AMO) and cefuroxime (CFU) were purchased from Sigma‐Aldrich (Singapore). The blank urine samples were collected from healthy children.

### 2.2 Equipment

The liquid chromatography system was a LaChrom Elite (Merck–Hitachi, Japan) composed of an autosampler L‐2200, 2 pumps L‐2130, a column Oven L‐2350 and a diode array detector (DAD) L‐2455. The system was controlled by EZchrom Elite version 3.18 HPLC System Manager Software (Merck–Hitachi, Japan). The analysis of CFD, CPL, CFO and CFI was performed on a 5 μm LichroCart^®^ Purospher Star reversed‐phase C_18_ end‐capped column (5 μm, 250 × 4.6 mm), equipped with a LichroCart^®^ C_18_ (5 μm, 4 × 4 mm) guard column (Merck, Darmstadt, Germany). A LichroCart^®^ Purospher Star reversed‐phase C_8_ end‐capped column (5 μm, 125 × 4.6 mm) equipped with a LichroCart^®^ C_8_ (5 μm, 4 × 4 mm) guard column was used to separate AMO and CFU. The SPE was performed on an Isolute^®^ strong cation exchange (SCX), 50 mg/ml 96‐fixed‐well plates for CFD, CPL, CFO and CFI, and Isolute^®^ C8 100 mg/ml 96 fixed well plates for AMO and CFU (Biotage AB, Uppsala, Sweden).

### 2.3 HPLC analytical conditions

The mobile phases consisted of a gradient mode comprising components A (20 mm KH_2_PO_4_ adjusted to pH 2.8 using H_3_PO_4_ for CFD, CPL, CFO and CFI, or 20 mm KH_2_PO_4_ adjusted to pH 3.0 for AMO and CFU) and B (pure ACN). The compositions of the mobile phases, flow rates, temperatures and wavelengths for detecting the analytes are presented in Table 1.

**Table 1:** Chromatographic conditions for six *β*‐lactams

Time (min)	Pump A (%)	Pump B (%)	Flow rate (ml/min)	Wavelength (nm)	Oven temperature (°C)
*AMO, CFU*	*ACN*	*KH*_*2*_ *PO*_*4*_ *20 mm, pH 3.0*			
0–3	5	95	1	229 (AMO)	30
3.1–10	16	84	1	273 (CFU)	
10.1–17	5	95	1		
*CFD, CPL, CFO, CFI*	*ACN*	*KH*_*2*_ *PO*_*4*_ *20 mm, pH 2.8*			
0–6	10	90	1.2	265 nm	40
6–8	14	86	1.4		
8–10.5	14	86	1.4		
10.6–14	25	75	1.4		
14.1–15	10	90	1.4		
15–18	10	90	1.2		

CFD, Cefadroxil; CPL, cephalexin; CFO, cefaclor; CFI, cefixime; AMO, amoxicillin; CFU, cefuroxime.

### 2.4 Preparation of standard solutions and quality controls

Stock solutions of CFD, CPL (10 mg/ml) and CFO (5 mg/ml) were prepared by dissolving the standards in water. CFI, AMO and CFU (2 mg/ml) were prepared by dissolving the standards in MeOH. The stock solutions were combined and further diluted with water to obtain fresh working solutions for calibration curves (CC) ranging from 3 to 300 μg/ml for CFD, CPL, CFO and CFI, and from 2 to 200 μg/ml for AMO and CFU. Quality control (QC) stocks and working solutions were independently prepared in the solvents described above. The concentrations of low, medium and high QC working solutions were 5, 100 and 250 μg/ml for CFD, CPL, CFO and CFI, and 4, 40 and 160 μg/ml for AMO and CFU, respectively.

Urine samples for CC determination were prepared by diluting (1:10, v/v) the respective working solutions with diluted blank urine (1:5, v/v with pure water) to give eight CC points at 0, 0.3, 0.6, 2.0, 6.0, 12.0, 20.0 and 30.0 μg/ml for CFD, CPL, CFO and CFI, and at 0, 0.2, 0.5, 1.0, 2.0, 5.0, 10.0 and 20.0 μg/ml for AMO and CFU. Three QC levels were prepared in the same way to give the low (QCL), medium (QCM) and high (QCH) concentrations of 0.5, 10.0 and 25.0 μg/ml for CFD, CPL, CFO and CFI, and 0.4, 4.0 and 16.0 μg/ml for AMO and CFU.

### 2.5 Sample preparation

Urine samples were thawed at room temperature for 20 min then mixed for 20 s before being centrifuged at 10,000***g*** for 5 min at room temperature. After 10 min rest on the bench, 50 μL urine samples were mixed with 200 μL water. The mixture was then added to 250 μL of 4% formic acid. The resulting mixture was mixed by vortexing for 15 s and then rested for 2 min prior to being centrifuged at 10,000 rpm for 4 min.

The mixed samples (500 μL) were then loaded into Isolute^®^ SCX, 50 mg/1 ml (CFD, CPL, CFO, and CFI) and Isolute^®^ C8 100 mg/1 ml (AMO and CFU), 96‐fixed‐well plates. SPE procedures are demonstrated in Table 2. Twenty microliters of each eluate was injected into an equilibrated HPLC system.

**Table 2:** Solid‐phase extraction (SPE) procedures for six *β*‐lactams

SPE step	Solvent	Volume (ml)	Flow rate (ml/min)	Equilibrium time (min)	Vacuum pressure (Bar)
*AMO, CFU*					
Condition	MeOH	1	2	—	—
	Formic acid 2%	1	1	—	—
Load	Sample	0.5	0.5	—	—
Wash	Water	0.5	1	—	−0.1
Elution	KH_2_PO_4_ 20 mm–MeOH (1:1, v/v)	0.25 × 2	1	2.00	−0.05
*CFD, CPL, CFO, CFI*					
Condition	MeOH	1	1–2	—	—
Equilibrate	Formic acid 2%	1	1–2	—	—
Load	Sample	0.5	0.5	—	—
Wash	Formic acid 2%	1	1	—	−0.1
	MeOH	0.5	1	—	−0.1
Elute	K_2_HPO_4_ 150 mm–MeOH (1:1, v/v)	0.25 × 2	0.5	2.00	−0.05

### 2.6 Method validation

A method validation of six *β*‐lactams in urine was performed for selectivity, carryover, sensitivity, linearity, recovery, precision and trueness in accordance with USA Food and Drug Administration (FDA) bioanalytical method validation guidelines (FDA, 2013).

### 2.7 Selectivity, carryover and sensitivity

Selectivity was evaluated by analyzing urine samples from six different healthy children and the chromatograms were evaluated for any sign of potential interferences with the drug identification and measurement. No interfering components were considered to be present when the signal was <20% of the limit of quantification (LLOQ) for analytes.

Carryover was evaluated by injecting two blank samples in the system after a duplicate of the highest calibration standard (upper limit of quantification, ULOQ) was run. The signal in the blank sample run after injection of the highest calibration standard should not be greater than 20% of the LLOQ.

Sensitivity was defined by the limit of detection (LOD) and the limit of quantification (LLOQ). The LOD was expressed as the concentration producing a signal three times higher than the noise from blank samples. The LLOQ was defined as the lowest concentration of analyte which could be determined with acceptable trueness and precision. The analyte signal of the sample with the LLOQ should be from 5 to 10 times higher than the baseline noise of six blank samples.

#### 2.7.1 Calibration curve and linearity

Linearity was assessed using the calibration curve consisting of eight calibration standards (including blank samples). For each antibiotic, calibration curves were obtained using the peak area of analytes (*y*) vs concentrations (*x*). The best regression models with or without data transformations and weighting factors were evaluated and selected using model options in HPLC System Manager Software‐EZChrom Elite version 3.18 (Hitachi‐Japan). This was intended to control the heteroskedasticity of our data and have the best predicted back‐calculated concentrations from unknown samples.

#### 2.7.2 Extraction recoveries

Antibiotic extraction recoveries were measured at three different levels (corresponding to the QC samples with low, medium and high concentrations) and determined by comparing the peak areas of the analytes spiked in urine after the extraction process with the peak area of an unprocessed standard solution prepared with identical concentrations.

#### 2.7.3 Precision and trueness

The intra‐day precision and trueness were evaluated at three different quality control levels (QCL, QCM and QCH) in four (CFD, CPL, CFO and CFI) and five (AMO and CFU) replicates on the same day. For inter‐day precision and trueness determinations, four replicates of QC levels on 3 days (CFD, CPL, CFO and CFI) and five replicates of QCs on four different days (AMO and CFU) were performed and evaluated. Intra‐ and inter‐assay precisions were calculated using single‐factor analysis of variance (ANOVA) and expressed as the mean relative standard deviation (RSD, %), which was equal to (SD/mean) × 100. Trueness error (%) was calculated as (% = [estimated concentration/nominal value]×100) by comparing the measured average concentration with the nominal concentrations at each QC level. Acceptance criteria were as follows: trueness error had to be within 85–115% of the nominal value, and intra‐ and inter‐precisions had to be <15%.

### 2.8 Method application on clinical samples

The urine samples were collected from pediatric patients who had a clinical diagnosis of ARIs (at least one of cough, sore throat, runny nose or nasal congestion as the chief complaint, lasting <5 days) at admission to the Children's Hospital 1 (CH1), Ho Chi Minh City, Viet Nam in 12 months. Parents or legal guardians consented to their children's participation. The protocol was approved by the Scientific and Ethics Committee of CH1, the Health Service of Ho Chi Minh City and the University of Oxford Tropical Research Ethics Committee (OxTREC 31–08). All of the samples were kept in the fridge and transferred to the Pharmacology Laboratory–Oxford University Clinical Research Unit, Ho Chi Minh City and frozen immediately at −80°C until assayed.

The clinical samples were analyzed and validated against freshly prepared CC and QC. The acceptance criteria on QC sets was <15% for precision and between 85 and 115% for trueness.

## 3 RESULTS AND DISCUSSION

### 3.1 Sample preparation and chromatographic separation

For sample treatment, the SPE procedures were established to extract six *β*‐lactams from urine samples. Four different sorbents (C_18_, C8, ABN and SCX supplied from Biotage) were used to process the samples. For CFD, CFO, CPL and CFI, the SCX sorbent was the best choice because it has double interaction mechanisms, cationic retention and a secondary nonpolar interaction, which help to facilitate maximum retention of the analytes. The recoveries were significantly improved using an organic solvent (MeOH) combined with high ionic strength buffer (di‐potassium hydrogen phosphate 150 mm) to elute these four compounds. AMO and CFU showed poor retentions on SCX. Also, there was a low recovery of AMO and the presence of interferences in the eluates after applying the SPE process using ABN sorbent. The recovery of AMO was low on C_18_ compared with C_8_ sorbent owing to its polar characteristics. Consequently, C_8_ nonpolar sorbent was chosen to extract AMO and CFU from urine samples with acceptable recoveries. The SPE procedures for six *β*‐lactams are presented in Table 2 and their recoveries are shown in Table 3.

**Table 3:** Precision, trueness and recovery of the HPLC methods for the determination of six *β*‐lactams in human urine samples (ANOVA)

		Concentration (μg/ml)	Precision (RSD)		Trueness (%)		Recovery (%)
			Inter‐day	Intra‐day	Inter‐day	Intra‐day	
CFD	QCL	0.5	3.59	7.61	102.7	100.7	94.40
(*n* = 4, *a* = 3)	QCM	10	6.09	8.59	93.35	90.76	92.90
	QCH	25	7.20	9.70	99.01	95.93	92.50
CFO	QCL	0.5	13.79	7.93	102.8	97.23	102.5
(*n* = 4, *a* = 3)	QCM	10	2.46	10.55	96.81	97.01	80.90
	QCH	25	10.53	10.75	99.17	104.6	76.20
CPL	QCL	0.5	2.35	5.12	100.5	101.8	94.90
(*n* = 4, *a* = 3)	QCM	10	5.77	7.41	100.1	101.7	93.40
	QCH	25	3.99	4.06	97.98	96.18	96.80
CFI	QCL	0. 5	12.08	9.13	102.0	96.23	90.60
(*n* = 4, *a* = 3)	QCM	10	9.71	5.75	97.21	93.76	89.20
	QCH	25	4.40	3.44	102.7	100.1	88.70
AMO	QCL	0. 4	7.25	8.32	93.91	95.70	65.87
(*n* = 5, *a* = 4)	QCM	4	4.62	5.98	101.1	98.88	90.87
	QCH	16	1.86	1.91	89.23	90.19	101.3
CFU	QCL	0.4	6.74	4.28	88.64	92.60	85.50
(*n* = 5, *a* = 4)	QCM	4	2.91	3.58	89.44	90.96	93.90
	QCH	16	3.27	2.42	85.87	86.81	98.24

Abbreviations: RSD, relative standard deviation (%); QCL, QCM, QCH, quality control at low, medium, high concentrations; *n*, number of samples in each run; *a*, number of runs.

**Figure 1:**
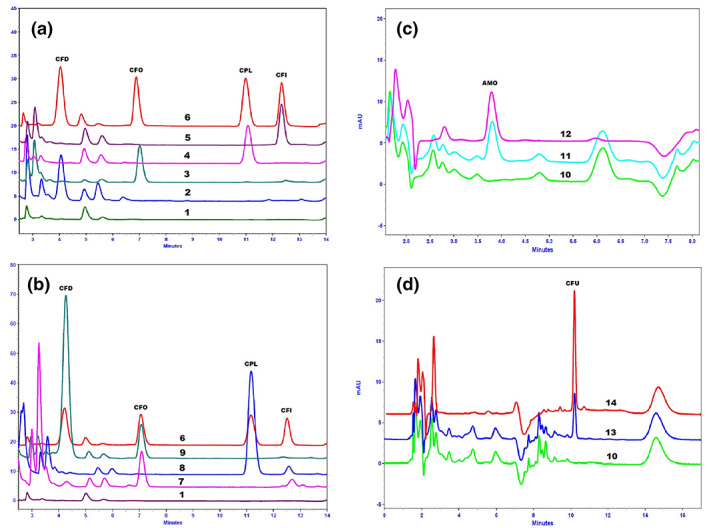
Chromatograms of six *β*‐lactams in urine samples. (a) Blank sample (1); patient samples with cefadroxil (CFD) (2), cefaclor (CFO) (3), cephalexin (CPL) (4) and cefixime (CFI) (5); spiked urine with four antibiotics (6) (12 μg/ml). (b) Blank sample (1); patient samples with three antibiotics: CFD, CFO and CFI (7); two antibiotics: CPL, CFI (8) and CFD, CFO (9); spiked urine with four antibiotics (6) (12 μg/ml). (c) Blank sample (10); patient sample with amoxicillin (AMO) (11); spiked urine with AMO (12) (10 μg/ml). (d) Blank sample (10); patient sample with cefuroxime (CFU) (13); spiked urine with CFU (14) (10 μg/ml).

In the development of chromatographic conditions for six *β*‐lactams, we intended to simultaneously analyze these antibiotics in one run. However, SCX, the best SPE sorbent for four *β*‐lactams (CFD, CPL, CFO and CFI), was not suitable for processing AMO and CFU, so we had to set up two separate extraction methods. Various reverse‐phase columns (Ascentis‐RP‐amide and Lichrospher RP_18_ end‐capped long columns for CFD, CFO, CPL and CFI, and LichroCart RP_18_ end‐capped, RP‐8 end‐capped 125 × 4 mm, 5 μm for AMO and CFU) were tested combining phosphate buffers at different pH (ranging from 2.8 to 5.0) and organic solvent (ACN). The best combination we found was a Lichrospher RP_18_ end‐capped 250 × 4.6 mm, 5 μm with KH_2_PO_4_ 20mm at pH 2.8–ACN for CFD, CFO, CPL and CFI, while the LichroCart RP_8_ end‐capped 125 × 4 mm, 5 μm with KH_2_PO_4_ 20 mm at pH 3.0–ACN was selected for AMO and CFU. The pH of the buffer was also found to have a significant influence on the analytical performance in terms of sensitivity. At high pH (4.0–5.0), the retention times of the analytes were shorter, but the separation between them and interferences was not satisfactory. Finally, the optimum values of pH, pH 2.8 and pH 3.0, were chosen for CFD, CFO, CPL and CFI and AMO, and CFU, respectively. Owing to the difference in polarities of the analytes, gradient programs of elution with different concentrations of ACN were applied to separate the analytes from matrix interferences. The compositions of the mobile phase, the gradients and flow rates, temperature and wavelength for detecting the analytes are presented in Table 1. The optimal wavelengths were 263, 260, 265 and 286 nm for CFD, CPL, CFO and CFI, respectively. The wavelength at 265 nm was chosen to detect these four antibiotics because their intensities were insignificantly changed in chromatograms at 265 nm compared with the optimal wavelength of each analyte. Wavelengths were set at 229 and 273 nm for AMO and CFU, respectively. The developed methods provided good resolutions and selectivity. The six *β*‐lactams were successfully separated from matrix contaminants with symmetrical peak shapes at the respective retention times. Figure 1 demonstrated the chromatographic separation of six *β*‐lactams, including an unknown sample, showing that the patient's samples were positive with at least one *β*‐lactam. Retention times (*t*_R_) in urine were 4.1, 6.9, 10.8, 12.2, 3.7 and 10.1 min for CFD, CFO, CPL, CFI, AMO and CFU, respectively.

### 3.2 Validation

The validation methods of six *β*‐lactams in human urine was performed to assess selectivity, sensitivity, recovery, linearity, precision and trueness in compliance with the FDA's bioanalytical method validation guidelines (FDA, 2013).

#### 3.2.1 Selectivity, carryover and sensitivity

No interference peaks were observed in the drug‐free human urine samples following sample pretreatment procedures for six *β*‐lactams. The analytes were well defined and separated from matrix contaminants, with symmetrical peak shapes at the respective retention time for the six *β*‐lactams. In all validation batches, no interferences from urine were found co‐eluating with analytes, thus ensuring the selectivity of the methods. This was also confirmed throughout the entire clinical sample analysis.

Injection of blank samples directly after injection of the ULOQ showed a signal <20% of the LLOQ for each antibiotic, thus satisfying the acceptance criteria for the carryover.

From 250 μL urine samples, the LODs were 0.08 μg/ml (for CFD, CPL, CFO and CFI), 0.1 μg/ml (AMO) and 0.05 μg/ml (CFU). The LLOQ was defined as the lowest concentration of analytes which had a signal‐to‐noise ratio of ≥5:1 and could be determined with acceptable precision and trueness (≤20%). It was determined as the lower limits of the calibration range (0.3 μg/ml for CFD, CPL, CFO and CFI and 0.2 μg/ml for AMO and CFU). Generally, these LLOQs were lower than or similar to those of previously reported HPLC‐UV methods ranging from 0.5 to 2.5 μg/ml for CFD, CPL, CFO and CFU (Denooz & Charlier, 2008, El‐Gindy et al., 2000, Kovach et al., 1991, McAteer et al., 1987, Wolff et al., 2013) and from 0.1 to 0.5 μg/ml for CFI (Bhinge et al., 2014; McAteer et al., 1987). Noticeably, some methods reported very high LLOQs for CPL (5 μg/ml; Najib et al., 1987) and CFD (12 μg/ml; Eshra et al., 1993) in urine samples. Regarding our method, for AMO, the LLOQ was lower than that mentioned in the previous published methods (varying from 0.4 to 5 μg/ml; Carlier et al., 2015; Colin et al., 2013; Legrand et al., 2016; Verdier et al., 2011).

#### 3.2.2 Calibration and linearity

The concentration range of the methods consisted of eight points (including blank urine). Methods were found to be linear over concentrations of 0, 0.3, 0.6, 2, 6, 12, 20 and 30 μg/ml (CFD, CPL, CFO and CFI) and 0, 0.2, 0.5, 1, 2, 5, 10 and 20 μg/ml (AMO and CFU). The calibration curve was determined by plotting the peak areas vs. the concentrations of analytes using regression analysis. The best models were quadratic, log–log transformed and 1/*x* weighting regression models (for CFD and CPD) and 1/*x* ^2^ regression models (for CFO and CFI). For AMO and CFU, linear regression with log–log transformation and nonweighting were the optimal models. The high coefficients of regression were achieved (*r* ^2^ > 0.99) for all tested *β*‐lactams.

#### 3.2.3 Recoveries

The mean recoveries of six *β*‐lactams in all QC levels are shown in Table 3. The lowest recovery was 65.87% for AMO and the highest was 102.5% for CFO.

#### 3.2.4 Precision and trueness

The precision and trueness of six *β*‐lactams, overall, were all satisfactory. The intra‐ and inter‐assay relative standard deviations were always <14%. The accuracies ranged from 85.87 to 102.78% at all QC levels for six *β*‐lactams in samples. The detailed results of the assay are shown in Table 3.

Even though the stability test was not performed as per the guidance instruction in our current HPLC methods, the stability of these antibiotics in different matrices was well established and confirmed in previous publications (Denooz & Charlier, 2008; El‐Gindy et al., 2000; Legrand et al., 2016; Nemutlu et al., 2009; Samanidou et al., 2003; Verdier et al., 2011). In addition, internal standards were not applied in this assay to correct any internal bias in the whole procedures. This could explain the significant variance seen at some levels. However, the precision and trueness were in fair agreement and satisfied the guidance.

### 3.3 Application to clinical samples

**Figure 2:**
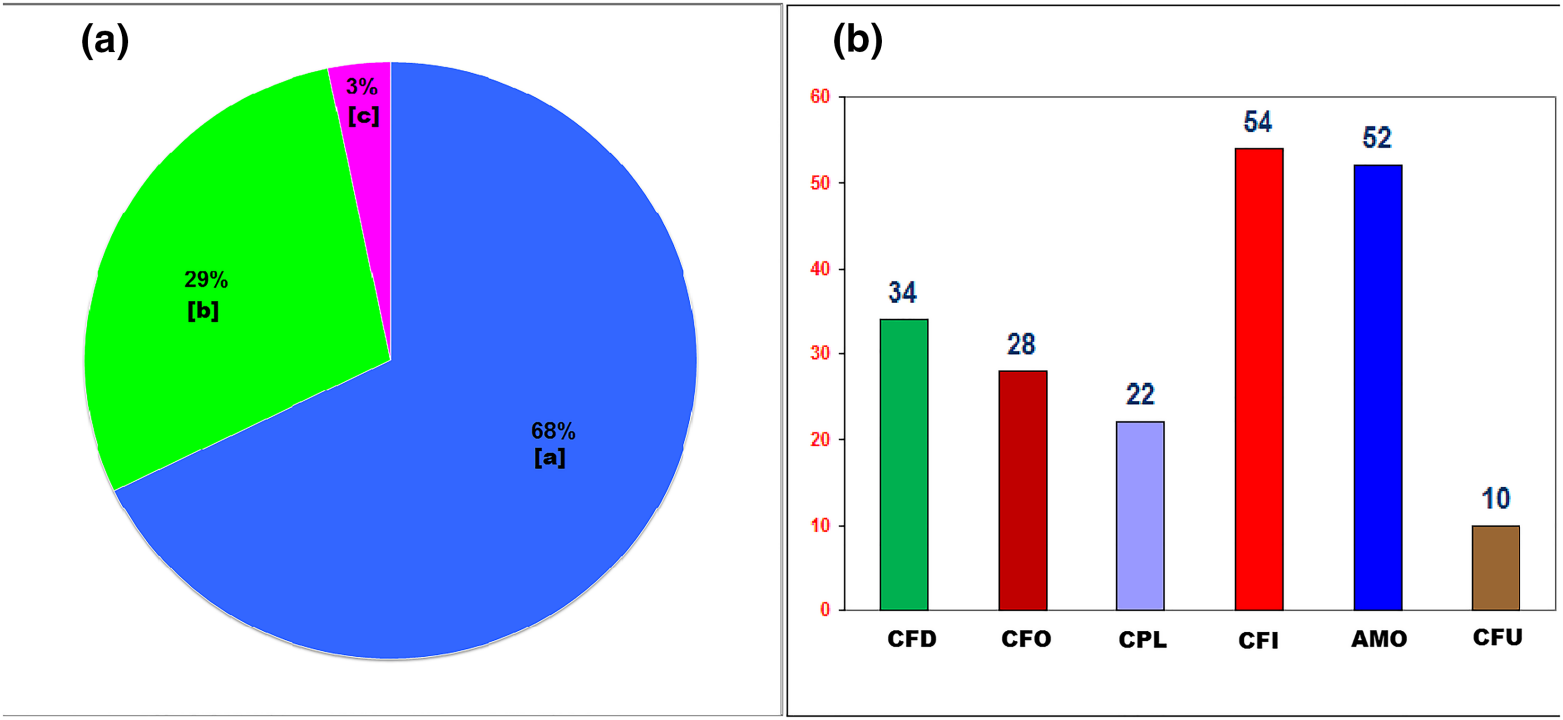
The determination of six *β*‐lactams in clinical urine samples. (a) Percentage of urine samples positive with at least one of six *β*‐lactam antibiotics tested. (a) negative; (b) positive with one antibiotic; (c) positive with more than one antibiotic (*N* = 553). (b) Frequency of *β*‐lactams that HPLC methods detected in urine samples of pediatric patients with ARIs.

The urine samples were collected from 563 pediatric patients under 16 years of age (50% of patients were less than 2 years old, and 95% of patients under 5 years old). The validated methods were successfully applied to determine the six *β*‐lactams in urine samples (10 patients with severe ARIs were anuria). Figure 2 presents the results of a qualitative measurement of the *β*‐lactams in clinical samples. Among of the tested *β*‐lactams, CFI was detected at the highest rate (54/553–9.8%), followed by amoxicillin (52/553–9.4%), while CFU was the least common identified medication, at only 1.8% (10/553).

The results demonstrated that 32.2% of patients had positive results with at least one out of the six investigated *β*‐lactam antibiotics. Our findings were similar to a study conducted in India, which also indicated that 31% of 64 children with febrile illness had antibiotics in the urine (Mathew et al., 2010). Of note, amoxicillin was found as the most frequently used antibiotic in the Indian study, at 17.2%, compared with cefixime (9.8%) in our study. However, both amoxicillin and cefixime were detected at the highest proportion and the same rate (8.8–25/286) in patients <2 years of age in our study. The difference might be due to the considerable disparity in the sample size as well as the kinds of antibiotics tested in the two studies. In the Indian study, the urine samples were assayed for five antibiotics including trimethoprim/sulfamethoxazole, amoxicillin, ciprofloxacin, cephalexin and cefuroxime while we tested for amoxicillin, cephalexin and cefuroxime and three other *β*‐lactams (cefadroxil, cefaclor and cefixime). Since other commonly used antibiotics (macrolides, fluoroquinolones and sulfamethoxazole/trimethoprim) were not tested by our current HPLC methods, we might assume that more than one‐third of patients with mild ARIs in our study had been given antibiotics prior to hospitalization.

As regulated by the Drug Administration of Vietnam, antibacterial agents must be purchased with a medical prescription (VNMOH, 2003). However, it has been shown that most antibiotics were easily obtained without prescription in the community drugstores of Vietnam (Hoa et al., 2009; Landers et al., 2010; Larsson et al., 2000; Llor & Cots, 2009). Our finding was consistent with this real situation and provided evidence that patients easily took the antibiotics without prescription for their fever illness. In our study, cefixime, a broad‐spectrum third‐generation cephalosporin, was the most commonly detected agent, accounting for 27% of tested antibiotics and about 9.8% (54/553) of enrolled patients. A major reason for dispensing broad‐spectrum antibacterial agents in the community setting might be due to inadequate antibiotic coverage by health workers in the pharmacies and/or private clinics (Hulscher, Grol, & Van Der Meer, 2010). In addition, 3.2% (18/553) of urine samples were positive with more than one *β*‐lactam antibiotic. Our findings demonstrated that patients might receive more than one antibiotic in a visit or they might visit multiple private clinics and/or pharmacies to obtain antibiotics without attempting to identify the causative organisms during their illness period. The overuse of broad‐spectrum cephalosporins and inappropriate combination of antibiotics raised a red alert on the misuse of antibiotics in Vietnam community settings. Antibiotics misuse increases drug adverse reactions and promotes the development of antimicrobial resistance, which is one of the most serious worldwide public health problems nowadays, especially in low‐ and middle‐income countries (Costelloe et al., 2010; Goossens et al., 2005; Karakonstantis & Kalemaki, 2019; Tilak, 2011). We suggest providing an intensive program of continuing medical/pharmacy education on the rational use of antibiotics combined with a supervision of antibiotic use in clinical practice. These are essential intervention steps, which should be given to the pharmacists and general practitioners in order to control the problem of inappropriate antibiotic use in Vietnam's community and clinical settings.

This is the first study, in which clinical samples (urine) were tested for determination of early antibiotic use in pediatric patients with ARIs in Vietnam. The accurate prevalence rate of early antibiotic use might be underestimated in this study because our current validated HPLC methods could not determine other commonly used antibiotics in patients with ARIs such as macrolides and fluoroquinolones. In addition, the clinical samples in our study were collected from patients aged less than 16 years old attending in only one pediatric hospital in Ho Chi Minh City, which might not represent the entire pediatric population in Vietnam. Moreover, the results of viral/microbial assays were also not interpreted together with the current findings of the detected *β*‐lactam antibiotics, which is beyond the scope of this article.

## 4 CONCLUSION

Two HPLC‐DAD methods were developed and validated to qualitatively determine six commonly used oral *β*‐lactams (CFD, CPL, CFO, CFI, AMO and CFU) in 553 urine samples obtained from pediatric patients with ARI. The broad‐spectrum third‐generation cephalosporin, cefixime, was frequently used and one‐third (178/553) of pediatric patients attending a pediatric hospital in Ho Chi Minh City of Vietnam, had taken at least one *β*‐lactam antibiotic for the self‐treatment of ARIs before hospital presentation. The study results further highlight and raise urgent warnings on antibiotic misuse in the Vietnam community setting. Continuing medical/pharmacy education programs should be regularly given to the general practitioners and pharmacists to ensure the rational use of antibiotics, contributing to control of antibiotic use and reducing antimicrobial resistance in the country.

## AUTHORS CONTRIBUTIONS

The study was conceived and designed by MDdJ, HRvD, TP and PVT, and the data and samples were collected by NNQM, PVT, KVD and PNP. The HPLC methods were developed and validated by PVT, TP, KVD and PNP. Clinical urine samples and data were analyzed by PVT, TP, KVD and PNP. The manuscript was written by PVT, KVD, TP and NNQM with review, comments and edit from all other authors. All authors have seen and agreed on the final manuscript prior to submission.

We have no conflicts of interest to declare.
